# Rice Seed Cultivar Identification Using Near-Infrared Hyperspectral Imaging and Multivariate Data Analysis

**DOI:** 10.3390/s130708916

**Published:** 2013-07-12

**Authors:** Wenwen Kong, Chu Zhang, Fei Liu, Pengcheng Nie, Yong He

**Affiliations:** 1 College of Biosystems Engineering and Food Science, Zhejiang University, 866 Yuhangtang Road, Hangzhou 310058, China; E-Mails: zjukww@163.com (W.K.); chuzh@zju.edu.cn (C.Z.); fliu@zju.edu.cn (F.L.); 2 Cyrus Tang Center for Sensor Materials and Applications, Zhejiang University, 866 Yuhangtang Road, Hangzhou 310058, China

**Keywords:** rice seed cultivar, hyperspectral imaging, random forest (RF), weighted regression coefficients (*B_W_*)

## Abstract

A near-infrared (NIR) hyperspectral imaging system was developed in this study. NIR hyperspectral imaging combined with multivariate data analysis was applied to identify rice seed cultivars. Spectral data was exacted from hyperspectral images. Along with Partial Least Squares Discriminant Analysis (PLS-DA), Soft Independent Modeling of Class Analogy (SIMCA), K-Nearest Neighbor Algorithm (KNN) and Support Vector Machine (SVM), a novel machine learning algorithm called Random Forest (RF) was applied in this study. Spectra from 1,039 nm to 1,612 nm were used as full spectra to build classification models. PLS-DA and KNN models obtained over 80% classification accuracy, and SIMCA, SVM and RF models obtained 100% classification accuracy in both the calibration and prediction set. Twelve optimal wavelengths were selected by weighted regression coefficients of the PLS-DA model. Based on optimal wavelengths, PLS-DA, KNN, SVM and RF models were built. All optimal wavelengths-based models (except PLS-DA) produced classification rates over 80%. The performances of full spectra-based models were better than optimal wavelengths-based models. The overall results indicated that hyperspectral imaging could be used for rice seed cultivar identification, and RF is an effective classification technique.

## 1. Introduction

Rice is one of the most common food crops in China and many other countries. The yield and quality of rice, which are mainly influenced by rice variety and growing conditions, are the biggest concerns of rice planting regions and consumers. Determination of rice seed variety and quality is the primary and essential step of rice planting [1]. In some cases, rice seed cultivars with good quality can be faked using poor quality cultivars or mistaken for other cultivars, which significantly affects the quality, yield and value of rice, thus, identification of rice seed cultivars is of great interest.

Different cultivars of rice show variations in size, shape, color and constitution, which cannot be accurately identified by human visualization. Traditional techniques used for rice variety identification like HPLC [2], or GC-MS [3] are time consuming and difficult to apply. Some new techniques such as machine vision and visible/near-infrared spectroscopy have been developed and applied to determine rice varieties. Machine vision is used to identify rice varieties based mainly on the morphology and texture [4,5]. Machine vision can capture image data of the entire sample, but the compositional detection is limited. Visible/near infrared spectroscopy has been proven to be efficient in determining rice quality and variety [6–9]. Visible/near-infrared spectroscopy acquires spectral information of a small area to get complete information on the chemical constituents, and spectral acquisition of the entire sample need repetitive measurement in different places of the sample which is always ignored, but no morphology or texture information is obtained. Machine vision and visible/near-infrared spectroscopy both acquire partial information about the sample, so a technique that acquires both spatial information and spectral information should be developed.

Hyperspectral imaging is an emerging technique combining the machine vision and spectroscopy techniques. Hyperspectral imaging acquires spatial images of samples at different wavelengths across regions of the electromagnetic spectrum (nowadays, UV-visible/near infrared region data is available), resulting in a three-dimensional hypercube. Hyperspectral imaging has recently gained popularity in many fields due to the spectral and spatial information it provides [10–12]. Hyperspectral imaging has been applied to identify some crop seed varieties and showed significant performance [13,14], but there are no studies using hyperspectral imaging to identify rice seed varieties.

Hyperspectral imaging acquires spectral and spatial information simultaneously, which provides a full dataset of internal and external features of samples. In hyperspectral imaging, spectral and spatial information is acquired from each pixel of the Region of Interest (ROI) in the image. The mean spectrum of all pixels in the ROI is used as the spectrum of the sample. Another advantage of hyperspectral imaging is the visualization of chemical images, which can map the spatial distribution of the chemical constituents in the sample and provide intuitive information.

The development of multivariate statistics techniques shows significant benefits and potential in spectroscopic techniques, including hyperspectral imaging. After spectra or hyperspectral image acquisition, data analysis has a direct effect on the performance. Principal Component Analysis (PCA) [15], Partial Least Squares Discriminant Analysis (PLS-DA) [16], Soft Independent Modeling of Class Analogy (SIMCA) [17], Linear Discriminant Analysis (LDA) [18], K-Nearest Neighbor Algorithm (KNN) [19], Artificial Neural Network (ANN) [20], Support Vector Machine (SVM) [21] and Least-squares Support Vector Machine (LS-SVM) [22] have all been used to deal with classification issues, and these methods have proved to be effective. In recent years, modern applied mathematics has offered some new methods. Random forest (RF) [23,24] is a classifier containing many decision trees, and each decision tree forms a classifier. RF shows better accuracy and reliability than a single classifier, and it has been proved efficient in classification issues in many fields. In this study, RF is used as a classification method for rice seed cultivar identification.

The objective of this study is to explore the feasibility of rice seed variety identification using hyperspectral imaging and multivariate data analysis. The specific objectives are as follows: (a) Develop a hyperspectral imaging system in the NIR region (874–1,734 nm) to acquire hyperspectral images of rice seeds. (b) Extract spectral information, and build classification models by multivariate analysis methods. (c) Select sensitive wavelengths most relevant to rice seed cultivar identification, and build multivariate analysis models.

## 2. Experimental Section

### 2.1. Sample Preparation

Four cultivars of rice seed were obtained from a local seed company for our experiments, including Zhongzheyou No.1, Zhongzheyou No.5, Zhongzheyou No.8 and Zhongzheyou No.86. These four rice seed cultivars were hybridized from other rice seed cultivars. The rice seed cultivars were produced in the same year to avoid any effect of seed age. The rice seeds were placed in Petri dishes with a diameter as 10 cm and height of 1 cm. The rice seeds in one Petri dish represented one sample, and 225 samples (56 samples of Zhongzheyou No.1, 56 samples of Zhongzheyou No.5, 55 samples of Zhongzheyou No.8 and 58 samples of Zhongzheyou No.86) were acquired.

### 2.2. Hyperspectral Imaging System

**Figure 1.**
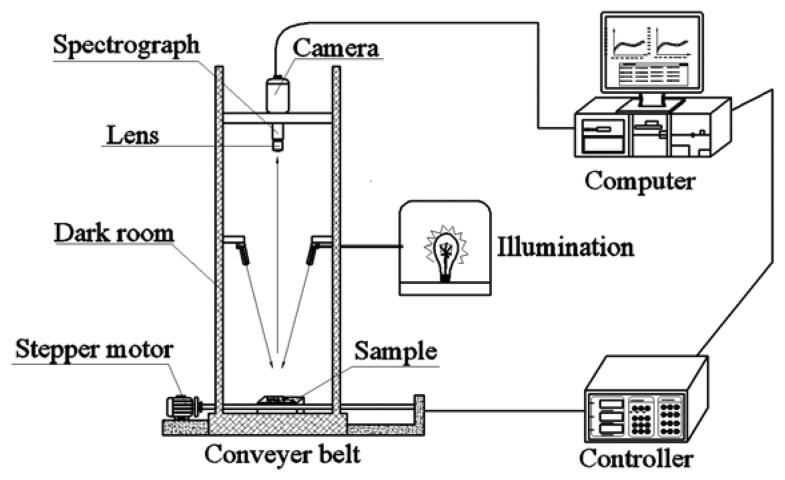
Hyperspectral imaging system.

Hyperspectral images of rice seeds were acquired by a laboratory-based hyperspectral imaging system shown in Figure 1. The developed system consists of an imaging spectrograph (ImSpector N17E; Spectral Imaging Ltd., Oulu, Finland) covering the spectral range of 874–1,734 nm with 256 bands, a high performance 320 × 256 camera (Xeva 992; Xenics Infrared Solutions, Leuven, Belgium), a camera lens (OLES22; Specim, Spectral Imaging Ltd.), two 150 W tungsten halogen lamps (Fiber-Lite DC950 Illuminator; Dolan Jenner Industries Inc., Boxborough, MA,USA) for illumination, a conveyer belt driven by a stepper motor (Isuzu Optics Corp., Taiwan, China), data acquisition and preprocessing software (Xenics N17E, Isuzu Optics Corp.), a computer and a darkroom. To acquire clear and non-deformed images, three parameters of this scheme are set, including the speed of movement of the conveyer belt, the exposure time of the camera and the height between the lens of camera and the sample, and these three parameters influence each other. In this study, as the height between the lens and the sample was set as 31 cm, the speed of the conveyer was set at 30 mm/s to ensure the same spatial shape of sample in the image and the exposure time was set as 3,000 μs to ensure a suitable light intensity. The samples were scanned in a line-scanning configuration. The acquisition of hyperspectral images includes the linear array scanning by the detector along the Y-axis and the moving of the sample on the X-axis. The images were saved as raw format.

### 2.3. Image Acquisition and Correction

In the laboratory-based NIR hyperspectral imaging systems, the Petri dishes filled with rice seeds were placed on the conveyer belt to be scanned line by line at a speed of 30 mm/s. A hyperspectral image was formed by 256 congruent gray scale sub-images representing the intensities of 256 wavelength bands. Thus, a 3D hypercube data representing the hyperspectral images contains the spectral and spatial information which could be used to identify rice seed cultivars.

Before hyperspectral image acquisition, white correction and dark correction were performed to acquire white (W) and dark (B) reference images. The acquisition of the dark reference image (B) is to remove the influence of dark current in the camera. The dark reference image (B) was acquired by turning off the light source together with covering the camera lens completely with its opaque cap while the white reference image (W) was acquired by using a white Teflon tile with nearly 100% reflectance.

Then the calibrated image (Ic) was calculated by using the raw hyperspectral image (Iraw), white reference image (W) and dark reference image (B) according to the following equation:

(1) $$Ic=\frac{\text{Iraw}-B}{W-B}$$

### 2.4. Spectra Data Exaction

After the hyperspectral images were corrected, the ROI with size of 15 × 15 pixels were plotted in the sample regions of images. The spectrum of each pixel was exacted, and the average spectrum of all pixels in the ROI was used as the spectrum of the sample. In total, 225 spectra of 225 samples were acquired.

### 2.5. Multivariate Analysis Methods

#### 2.5.1. Principal Component Analysis

Principal Component Analysis [15] is a data description and dimension reduction method which is widely used to deal with large datasets like spectral data. The large datasets are transformed into a small number of uncorrelated variables (called Principal Components, PCs). Each PC is a linear combination of the original data, and the number of PCs is as many as the original variables. The first few PCs could explain most of the sample data, which results in the data dimension reduction. PCA could reveal the variables that determine some inherent structure in the data, which could be interpreted in chemical or physico-chemical terms. Scores scatter plot of PC1 and PC2 shows the most significant variability among samples.

#### 2.5.2. Partial Least Squares-Discriminant Analysis

Partial Least Squares-Discriminant Analysis [16] is a discriminant technique based on PLS regression (PLSR). Unlike PLSR, the response variable Y in PLS-DA is a set of dummy variables representing the classes of the samples (In this study, 1 for Zhongzheyou No. 1, 2 for Zhongzheyou No. 5, 3 for Zhongzheyou No. 8 and 4 for Zhongzheyou No. 86). The predicted value in PLS-DA is a real number, but not a dummy integer. Thus, a cut off value needs to be set to determine which class the sample belongs to. Generally, the cut off value is set as 0.5. PLS-DA is calculated with full cross validation methods.

#### 2.5.3. Soft Independent Modeling of Class Analogy

Soft Independent Modeling of Class Analogy [17] is a supervised discriminant analysis method based on PCA. For each class, a PCA model is built and then the residual variance of the modeled class with the residual variance of the unknown sample is compared to determine which category the sample belongs to. The number of PCs used in each class should be selected to achieve the best classification results.

#### 2.5.4. K-Nearest Neighbor Algorithm

K-Nearest Neighbor Algorithm [19] is a classification method based on the closest training examples in the feature space. If the majority of an unknown sample's K-nearest neighbors in training set belong to a certain class, then this unknown sample is classified as this class. The parameter K influences the performance of KNN model. The Euclidean distance is the most common algorithm used in KNN.

#### 2.5.5. Support Vector Machine

Support Vector Machine [21] is a widely used supervised statistical learning algorithm. SVM shows advantages in dealing with small sample, non-linear and high dimensional data. SVM is based on the structural risk minimum (SRM) and SVM has high generalization capacity and could provide a flexible and easy-to-compute solution. Selection of kernel function in SVM models has significant influence in model performance, and in this study, the commonly used Radial Bias Function (RBF) is used as kernel function. The regularization parameter *c*, which controls trade-off between the minimum training error and minimum model complexity, along with the kernel parameter *g* of the kernel function, which represents the width of the kernel function and reflects the degree of generalization are determined by a grid-search procedure in SVM.

#### 2.5.6. Random Forest

Random Forest [23,24] is a novel machine learning algorithm combining Breiman's ‘bagging’ idea and Ho's “random subspace method”. A RF classifier contains many decision trees, and each tree is grown from a bootstrap sample of the response variable. The best split is selected from a random subset of variables at each node of the tree, and then grows the tree to the maximum extent without pruning. Prediction can be made from new data by aggregating the outputs of all trees. RF is effective and fast to deal with a large amount of data. RF has shown the advantages to reduce variance and achieve comparable classification accuracy.

### 2.6. Wavelength Selection

Optimal wavelength selection is widely applied in spectral data analysis due to the redundancy and colinearity of spectral data. Besides, optimal wavelength selection helps to reduce dimension and build simple, stable and practical calibration models. The wavelengths carrying the most useful information could be selected as optimal wavelengths. Many variable selection methods have been applied in spectral data analysis. In this study, weighted regression coefficients of PLS-DA model are used to select optimal wavelengths [25,26]. The peaks and valleys in weighted regression coefficients plot with absolute value over a certain cutoff value are selected as optimal wavelengths, and the remaining wavelengths are thought to carry little or no information. Precondition for wavelength selection by weighted regression coefficients is the good performance of PLS-DA models. Hyperspectral images were analyzed by ENVI 4.6 (ITT, Visual Information Solutions, Boulder, CO, USA), and multivariate data analysis were applied on Unscrambler^®^ 10.1 (CAMO AS, Oslo, Norway) and Matlab R2009b (The Math Works, Natick, MA, USA).

## 3. Results and Discussion

### 3.1. Spectral Features of Rice Seeds

**Figure 2.**
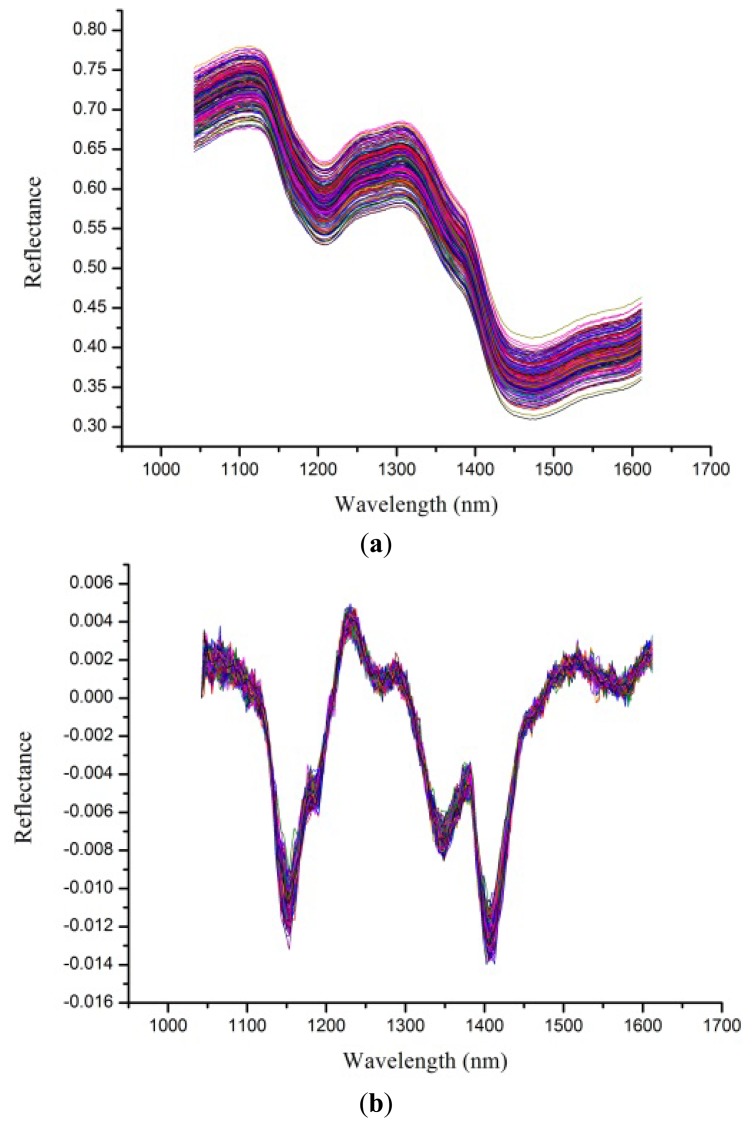
(a) Raw spectra of rice seeds. (b) first derivative preprocessed spectra of rice seeds.

Despite the fact the hyperspectral images were corrected before analysis, noises still existed. To avoid obvious noises, spectra from 1,039 to 1,612 nm (bands 50 to 220) were used for analysis. Raw spectral profiles of the four rice seed cultivars are shown in Figure 2a. The noises in the spectral range from 1,039 to 1,612 nm could still influence the performance of classification models, so a first derivative (1-Der) was applied to preprocess the raw spectra, and the resulting preprocessed spectral profiles are shown in Figure 2b. The 1-Der preprocessed spectra could be seen to retain and strengthen the information of the raw spectra. Spectral curves in Figure 2a,b showed overlaps and no obvious differences which indicated rice seeds could not be identified directly from these two figures. To solve this problem, classification models based on chemometrics were developed.

### 3.2. Multivariate Data Analysis

#### 3.2.1. PCA Analysis

**Figure 3.**
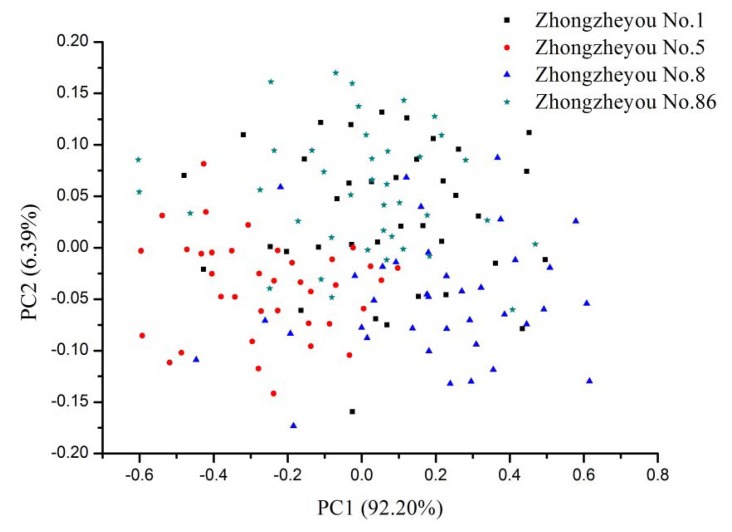
Scores scatter plot of PC1 and PC2 of raw spectra.

The advantage of PCA is to use a few PCs to represent original data. Generally, the first two PCs could explain most of the variance. In this study, PCA was performed on the raw spectra of calibration set with full cross validation. PC1 explained 92.20% variance and PC2 explained 6.39% variance, PC1 and PC2 explained 98.59% of the total variance. Figure 3 shows the scores plot of the NIR spectra from four rice seed cultivars. No clear separation between different cultivars was observed, and samples were distributed all along the space and overlapped. The scores plot indicated that global classification models could be built for rice seed cultivar identification.

#### 3.2.2. Multivariate Data Analysis Based on Full Spectra

Classification models were built based on the preprocessed spectra. The reliability of classification models was on the basis of classification accuracy. The classification results of PLS-DA, SIMCA, KNN, SVM and RF are shown in Table 1.

As seen from Table 1, the rice seed cultivars were accurately identified. The PLS-DA model and KNN model showed relatively low effectiveness, with classification accuracy of prediction of more than 80%. SIMCA, SVM and RF models obtained classification rates of 100% in both the calibration set and prediction set, which indicated that rice seed cultivars could be accurately identified. Among all four cultivars, Zhongzheyou No. 5 and Zhongzheyou No. 86 were well identified in all classification models, misclassification of Zhongzheyou No. 5 and Zhongzheyou No. 86 occurred only in the PLS-DA model with at most three misclassified samples. The misclassification rate of Zhongzheyou No. 1 in the PLS-DA model was over 10%, and the misclassification rate of Zhongzheyou No. 86 in the KNN model was also over 10%. In all, SIMCA, SVM and RF were deemed the optimal methods for rice seed cultivar identification.

#### 3.2.3. Optimal Wavelength Selection

**Figure 4.**
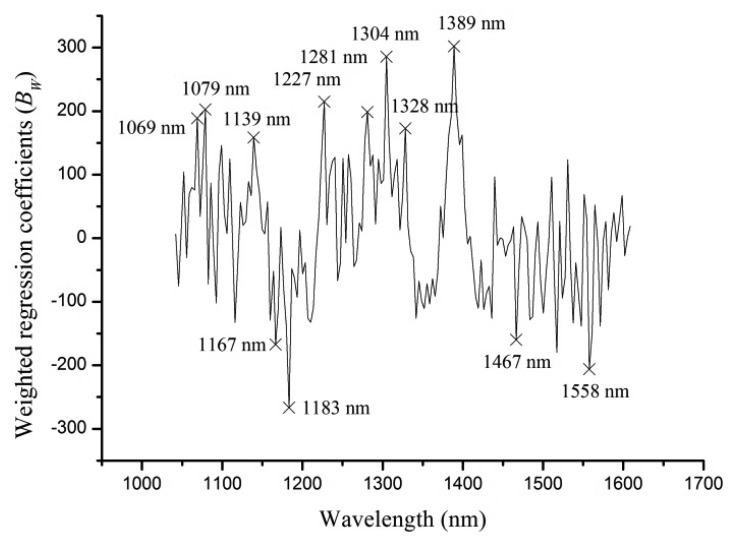
Weighted regression coefficients of PLS-DA model with selected wavelengths.

The full spectra contained 171 variables, and it was necessary to select sensitive wavelengths which carried more information to reduce dimension, computation complexity and simplify the calibration models. Weighted regression coefficients (*B_W_*) of PLS-DA model (shown in Figure 4) were used to select optimal wavelengths in this study. Strong peaks and valleys of *B_W_* with absolute value over 150 were selected as optimal wavelengths. As seen from Figure 4, 12 optimal wavelengths (1,069, 1,079, 1,139, 1,167, 1,183, 1,227, 1,281, 1,304, 1,328, 1,389, 1,467, 1,558 nm) were selected.

#### 3.2.4. Multivariate Data Analysis Based on Optimal Wavelengths

As a consequence of optimal wavelength selection, the selected 12 wavelengths were used as inputs of PLS-DA, KNN, SVM and RF. The performances of these models are shown in Table 2. PLS-DA model obtained the worst result, 45 samples out of 150 samples in calibration set and 25 samples out of 75 samples in prediction set were misclassified, with classification accuracy of 70% and 66.67%, respectively. Classification rates of KNN, SVM and RF models in the calibration set and the prediction set were all over 80%. The KNN model obtained the highest classification accuracy of 90.67% in the prediction set and the RF model obtained highest classification accuracy of 100% in the calibration set. Considering the prediction performance, KNN was the best classification model based on the optimal wavelengths. Zhongzheyou No. 1, and Zhongzheyou No. 8 showed poor prediction accuracy in all models. This result is similar to full spectra based models, which implied that Zhongzheyou No. 1 and Zhongzheyou No. 8 were difficult to identify, and appropriate models could help to obtain more accurate identification.

#### 3.2.5. Comparison of Full Spectra Based and Optimal Wavelengths Based Models

As seen from Tables 1 and 2, full spectra-based classification models gave better performances than optimal wavelengths-based models. Since SIMCA is a classification method based on PCA, it was not used to build models based on optimal wavelengths. The optimal wavelengths-based PLS-DA model showed the poorest performance in all models. The performance of PLS-DA models showed a sharp decline from full spectra to optimal wavelengths. Meanwhile, the performances of KNN, SVM and RF showed little changes in the two cases. Optimal wavelengths-based models used 12 wavelengths instead of 171 wavelengths, i.e., the number of wavelengths was decreased 92.98%, and performances of models decreased 16% at most in both calibration set and prediction set (except in the PLS-DA models). On the other hand, optimal wavelengths-based models gave a classification accuracy over 80% (except the PLS-DA model), which indicated that optimal wavelengths could be used for rice seed cultivar identification. In all, the full spectra-based SIMCA, SVM and RF models obtained 100% accuracy in the calibration set and the prediction set, which implied that full spectra would be better for rice cultivar identification than optimal wavelengths selected by weighted regression coefficients. Generally, wavelength selection had the advantage in improving the performance, and many wavelength selection methods have been applied in spectral data analysis. In this study, optimal wavelengths obtained reliable but not very reliable performances. For further study, different wavelength selection methods would be applied to explore the best one for rice seed cultivar identification.

## 4. Conclusions

NIR hyperspectral imaging combined with multivariate data analysis was applied to identify rice seed cultivars. Spectral information was exacted from hyperspectral images of rice seeds, and different classification models were built. PLS-DA, SIMCA, KNN, SVM and RF models based on full spectra obtained good performance, and the SIMCA, SVM and RF models showed 100% classification rates in both the calibration set and prediction set. Optimal wavelengths were selected based on the weighted regression coefficients, and PLS-DA, KNN, SVM and RF classification models were built. The results showed that the optimal wavelengths-based PLS-DA model gave the worst performance with a classification accuracy lower than 80%. Full spectra-based models performed better than optimal wavelengths-based models, RF as a classification methods showed good performance like the KNN, SIMCA and SVM models. The overall results indicated that it was feasible to use hyperspectral imaging for rice seed cultivar identification, and that RF was an effective modeling method.

## Figures and Tables

**Table 1.** Results of classification models based on full spectra.

**Model**		**1**		**2**		**3**		**4**		**Total**	

		a **Nr/Nt**	b **accu**	**Nr/Nt**	**accu**	**Nr/Nt**	**accu**	**Nr/Nt**	**accu**	**Nr/Nt**	**accu**
PLS-DA	c Cal	32/37	86.49%	36/37	97.30%	37/37	100%	38/39	97.44%	143/150	95.33%
	d Pre	13/19	68.42%	17/19	89.47%	17/18	94.44%	16/19	84.21%	63/75	84.00%
SIMCA	Cal	37/37	100%	37/37	100%	37/37	100%	39/39	100%	150/150	100%
	Pre	19/19	100%	19/19	100%	18/18	100%	19/19	100%	75/75	100%
KNN	Cal	34/37	91.89%	37/37	100%	30/37	81.08%	39/39	100%	140/150	93.33%
	Pre	17/19	89.47%	19/19	100%	13/18	72.22%	19/19	100%	68/75	90.67%
SVM	Cal	37/37	100%	37/37	100%	37/37	100%	39/39	100%	150/150	100%
	Pre	19/19	100%	19/19	100%	18/18	100%	19/19	100%	75/75	100%
RF	Cal	37/37	100%	37/37	100%	37/37	100%	39/39	100%	150/150	100%
	Pre	19/19	100%	19/19	100%	18/18	100%	19/19	100%	75/75	100%

a Nr is the number of rightly classified samples; Nt is the total number of samples.

b accu is the classification accuracy.

c Cal represents the calibration set of the samples.

d Pre represents the prediction set of the samples.

**Table 2.** Results of classification models based on optimal wavelengths.

**Model**		**1**		**2**		**3**		**4**		**Total**	

		a**Nr/Nt**	b**accu**	**Nr/Nt**	**accu**	**Nr/Nt**	**accu**	**Nr/Nt**	**accu**	**Nr/Nt**	**accu**
PLS-DA	c Cal	20/37	54.05%	26/37	70.27%	28/37	75.68%	31/39	79.49%	105/150	70%
	d Pre	11/19	57.89%	15/19	78.95%	10/18	55.56%	14/19	73.68%	50/75	66.67%
KNN	Cal	36/37	97.30%	34/37	91.89%	32/37	86.49%	38/39	97.44%	140/150	93.33%
	Pre	17/19	89.47%	19/19	100%	13/18	72.22%	19/19	100%	68/75	90.67%
SVM	Cal	37/37	100%	36/37	97.30%	36/37	97.30%	37/39	94.87%	146/150	97.33%
	Pre	17/19	89.47%	16/19	84.21%	16/18	88.89%	18/19	94.74%	67/75	89.33%
RF	Cal	37/37	100%	37/37	100%	37/37	100%	39/39	100%	150/150	100%
	Pre	17/19	89.47%	15/19	78.95%	13/18	72.22%	18/19	94.74%	63/75	84%

a Nr is the number of rightly classified samples; Nt is the total number of samples.

b accu is the classification accuracy.

c Cal represents the calibration set of the samples.

d Pre represents the prediction set of the samples.
